# Mechanisms underlying the postexercise baroreceptor‐mediated suppression of heat loss

**DOI:** 10.14814/phy2.12168

**Published:** 2014-10-07

**Authors:** Ryan McGinn, Gabrielle Paull, Robert D. Meade, Naoto Fujii, Glen P. Kenny

**Affiliations:** 1 Human and Environmental Physiology Research Unit, School of Human Kinetics, University of Ottawa, Ottawa, Canada

**Keywords:** Baroreflex, postexercise, skin blood flow, sudomotor, thermoregulation

## Abstract

Reports indicate that postexercise heat loss is modulated by baroreceptor input; however, the mechanisms remain unknown. We examined the time‐dependent involvement of adenosine receptors, noradrenergic transmitters, and nitric oxide (NO) in modulating baroreceptor‐mediated changes in postexercise heat loss. Eight males performed two 15‐min cycling bouts (85% VO_2max_) each followed by a 45‐min recovery in the heat (35°C). Lower body positive (LBPP), negative (LBNP), or no (Control) pressure were applied in three separate sessions during the final 30‐min of each recovery. Four microdialysis fibres in the forearm skin were perfused with: (1) lactated Ringer's (Ringer's); (2) 4 mmol·L^−1^ Theophylline (inhibits adenosine receptors); (3) 10 mmol·L^−1^ Bretylium (inhibits noradrenergic transmitter release); or (4) 10 mmol·L^−1^ l‐NAME (inhibits NO synthase). We measured cutaneous vascular conductance (CVC; percentage of maximum) calculated as perfusion units divided by mean arterial pressure, and local sweat rate. Compared to Control, LBPP did not influence CVC at l‐NAME, Theophylline or Bretylium during either recovery (*P *>**0.07); however, CVC at Ringer's was increased by ~5‐8% throughout 30 min of LBPP during Recovery 1 (all *P *<**0.02). In fact, CVC at Ringer's was similar to Theophylline and Bretylium during LBPP. Conversely, LBNP reduced CVC at all microdialysis sites by ~7–10% in the last 15 min of Recovery 2 (all *P *<**0.05). Local sweat rate was similar at all treatment sites as a function of pressure condition (*P *>**0.10). We show that baroreceptor input modulates postexercise CVC to some extent via adenosine receptors, noradrenergic vasoconstriction, and NO whereas no influence was observed for postexercise sweating.

## Abstract (precis)

To assess the mechanisms of the baroreceptor‐mediated suppression of cutaneous blood flow and sweating postexercise, eight young men performed two 15‐min bouts of cycling at 85% of their VO_2max_ each followed by 45 min of recovery during which positive, negative, or no pressure were applied to the lower limbs. Baroreceptors modulated cutaneous blood flow via nitric oxide (panel B), adenosine receptor (panel C), and noradrenergic vasoconstrictor (panel D) dependent mechanisms. On the other hand, baroreceptors were not shown to modulate postexercise sweating.

## Introduction

A growing body of empirical evidence indicates that postexercise thermoregulatory control is altered in humans. The cessation of exercise is associated with a suppression of heat loss responses (i.e., cutaneous blood flow and sweating) such that only ~50% of the heat gained during exercise is lost during the first hour of recovery (Kenny et al. 2008). It is believed that nonthermal factors of central origin are involved in this response (Kenny and Journeay 2010) including a baroreceptor‐mediated suppression of heat loss. Specifically, previous work has indicated that baroreceptor loading status has important implications in the control of cutaneous blood flow during passive heating at rest (Kellogg et al. 1990; Crandall et al. 1996), during exercise (Johnson and Park 1981; Mack et al. 1995, 2001), and in the postexercise period (Kenny et al. 2003a; Journeay et al. 2004; Gagnon et al. 2008). Similarly, a baroreceptor‐mediated modulation of sweating has been reported (Mack et al. 1995; Kenny et al. 2003b; Journeay et al. 2004); however, it has not been consistently observed across all studies (Mack et al. 2001; Wilson et al. 2001, 2005). In addition, some findings indicate that a greater magnitude of hyperthermia reduces the baroreceptor‐mediated modulation of cutaneous blood flow and sweating following exercise (Gagnon et al. 2008). However, there remains no information regarding the mechanisms by which the baroreceptors modulate postexercise heat loss at the level of the end‐organ (i.e., skin vessels and/or sweat gland).

Heat stress is associated with substantial increases in cutaneous blood flow mediated initially by the removal of tonic vasoconstrictor activity followed by the onset of cutaneous active vasodilation (Grant and Holling 1938; Johnson and Proppe 1996). The mechanism(s) by which cutaneous blood flow is suppressed following exercise remains to be fully elucidated; however, a recent study has implicated the time‐dependent involvement of adenosine receptors and noradrenergic vasoconstriction (McGinn et al. 2014). In particular, inhibition of adenosine receptors reportedly sustained cutaneous blood flow above a control skin site for the entire recovery (60 min) whereas inhibiting noradrenergic vasoconstriction was found to attenuate the suppression in cutaneous blood flow during the first 30 min postexercise. However, it was unclear whether these observations were the resultant effector response mediated by central baroreceptor input. Conversely, inhibition of nitric oxide production did not substantially alter cutaneous blood flow, albeit a role could not be discounted (McGinn et al. 2014). In fact, given that nitric oxide remains one of the most prominent active vasodilator substances (Kellogg et al. 1998; McCord et al. 2006; Stanhewicz et al. 2012; Wong 2013) and that changes in shear stress (indicated by changes in blood flow and/or pressure) can modulate nitric oxide production (Kuchan and Frangos 1994; Vequaud and Freslon 1996), it is plausible to suggest that changes in baroreceptor loading status during the postexercise period may impair nitric oxide production, and thereby cutaneous blood flow. On the other hand, sweating is the major avenue for heat loss during exercise in the heat, and is primarily mediated by cholinergic nerves (Shibasaki and Crandall 2010; Machado‐Moreira et al. 2012). In addition, nitric oxide has been shown to mediate sweating during exercise‐induced heat stress (Welch et al. 2009; Stapleton et al. 2014). A study by Stapleton et al. (2014) employed intermittent exercise (at ~52% of maximal oxygen consumption) and found that the role of nitric oxide in sweating was observed to a similar extent with each subsequent exercise bout. In contrast, a role for nitric oxide during each recovery period was not evident. As with cutaneous blood flow, it is plausible to suggest that baroreceptor unloading associated with postexercise hypotension (i.e., a decrease in shear stress) may inhibit nitric oxide production, and thereby suppress sweating during the postexercise period.

The purpose of this study was to evaluate the mechanisms by which baroreceptor loading status modulates the suppression in heat loss during recovery from exercise in the heat. High intensity intermittent exercise (2 bouts) in the heat was incorporated into this study to achieve progressively greater levels of hyperthermia. Furthermore, we induced changes in baroreceptor loading status during each postexercise period through the application of lower body positive pressure (LBPP) or negative pressure (LBNP) as commonly employed (Kellogg et al. 1990; Mack et al. 1995, 2001; Crandall et al. 1996; Journeay et al. 2004). We hypothesized that compared to a Control condition: (1) LBPP would increase cutaneous blood flow and sweating via nitric oxide‐dependent mechanisms whereas LBNP would only suppress cutaneous blood flow to a greater extent; (2) changes in baroreceptor loading status would also modulate cutaneous blood flow via noradrenergic vasoconstrictor activity and adenosine receptor activation evidenced by similar responses between pressure conditions; and (3) that the baroreceptor influence of cutaneous blood flow and sweating would be attenuated at greater levels of hyperthermia.

## Materials and Methods

### Ethical approval

The current experimental protocol was approved by the University of Ottawa Health Sciences and Science Research Ethics Board and was in accordance with the Declaration of Helsinki. Written informed consent was obtained from all volunteers prior to their participation in the study.

### Participants

Eight young males from the university community volunteered for the current study. Participants were healthy, non‐smoking, and normotensive with no history of respiratory, metabolic, or cardiovascular disease. Age, height, body mass, body surface area, and maximal oxygen consumption for the participants were (mean ± standard deviation): 23 ± 4 years, 177 ± 7 cm, 81 ± 9 kg, 1.98 ±0.14 m^2^, and 49 ± 5 mL·kg^−1^·min^−1^, respectively. All participants reported being physically active (≥30 min of structured moderate‐to‐vigorous physical activity for at least three sessions per week).

### Experimental design

Participants volunteered for one screening visit and three experimental sessions. For both the screening visit and the experimental sessions, participants were asked to abstain from alcohol, caffeine, and severe or prolonged physical activities for at least 24 h. Participants were instructed to eat a small breakfast (i.e., toast and orange juice) 2 h prior to arriving at the laboratory and to drink 500 mL of water the night before as well as the morning of each session. All experimental sessions were performed in a randomized order, started between 0830 h and 1030 h (at the same time for a given participant) and were separated by at least 72 h.

The screening visit consisted of measurements of body height and mass as well as maximal oxygen consumption. Body height was determined using a stadiometer (Detecto, model 2391, Webb City, MO), while body mass was determined using a digital high‐performance weighing terminal (model CBU150X, Mettler Toledo Inc., Mississauga, ON, Canada). Body surface area was subsequently calculated from the measurements of body height and mass (Du Bois and Du Bois 1916). Maximum oxygen consumption was determined by indirect calorimetry (MCD Medgraphics Ultima Series, Sun Tech Medical, Morrisville, NC) during a progressive incremental exercise protocol performed on an upright seated constant‐load cycle ergometer (Corival, Lode B.V., Groningen, the Netherlands). The participants were instructed to pedal at a cadence of 80–90 r.p.m. at 100 W for the first minute and the external workload was increased by 20 W every minute thereafter until the required cadence could no longer be maintained.

Upon their arrival for the experimental sessions, participants provided a urine sample and voided their bladder before a nude body mass measurement. Urine specific gravity was assessed using a refractometer (Reichert TS 400 total solids refractometer, Reichert Inc., Depew, NY). The participants then rested in the semi‐recumbent position on a bed for a 30 min instrumentation period at an ambient room temperature of 25°C and 30% relative humidity. Four microdialysis fibres (30 kDa cutoff; MD 2000, Bioanalytical Systems Inc., West Lafayette, IN) were inserted in the dermal layer of the skin on the right dorsal forearm under aseptic conditions with each fibre separated by at least 4 cm. A 25 gauge needle was first inserted into the dermal space of the skin with entry and exit points ~25 mm apart. The microdialysis fibre was then threaded through the lumen of the needle which was subsequently withdrawn to leave a 10 mm dialysis membrane in the dermal space (Bioanalytical Systems Inc.). Following instrumentation, all microdialysis fibres were first perfused with lactated Ringer's solution (Baxter, Deerfield, IL) at a rate of 2 *μ*L·min^−1^ via a micro perfusion pump (model 400, CMA Microdialysis, Solna, Sweden) for at least 60 min to allow for the resolution of the local hyperemic response.

The participants were then moved to an environmental chamber regulated at 35°C and 20% relative humidity and were seated in the upright position. At this point, each microdialysis site was instrumented for the simultaneous measurement of local sweat rate and cutaneous blood flow (see below for details). During this time, the microdialysis fibres were perfused at a rate of 2 *μ*L·min^−1^ with either: (1) lactated Ringer's solution, serving as the control skin site (Ringer's); (2) 10 mmol·L^−1^ *N*^*G*^‐nitro‐l‐arginine methyl ester (Sigma‐Aldrich, St Louis, MO), a non‐selective inhibitor of nitric oxide synthase and thus nitric oxide production (l‐NAME); (3) 4 mmol·L^−1^ theophylline (Sigma‐Aldrich), a non‐selective competitive adenosine (A_1_/A_2_) receptor inhibitor (Theophylline); and (4) 10 mmol·L^−1^ bretylium tosylate (Finetech Industry Limited, London, UK), an inhibitor of the presynaptic release of vasoconstrictor neurotransmitters (e.g., noradrenaline and neuropeptide Y) (Bretylium). These concentrations were chosen based on those used in previous studies employing intradermal microdialysis for l‐NAME (Minson et al. 2001; Fieger and Wong 2010, 2012; McGinn et al. 2014; Swift et al. 2014), Theophylline (Fieger and Wong 2010, 2012; McGinn et al. 2014; Swift et al. 2014), and Bretylium (Wilson et al. 2004; McGinn et al. 2014). Drug infusion was maintained for 45 min to ensure the establishment of each blockade (Minson et al. 2001; Wilson et al. 2004; Fieger and Wong 2010).

Prior to the baseline period, a cold‐pressor test was performed by immersing the left hand into an ice bath for ~3 min in order to verify that Bretylium would block reflex vasoconstriction observed by comparison to the Ringer's site (Pergola et al. 1994). Once cutaneous blood flow had returned to resting levels, 10 min of baseline data collection ensued. Thereafter, participants were transitioned to an upright cycle ergometer (Corival, Lode B.V.) and performed 15 min of exercise at 85% of their pre‐determined maximal oxygen consumption (Exercise 1). Immediately after the cessation of cycling, participants were moved to a pressure box sealed at the waist (<5 min) and rested for 45 min of recovery in the upright seated posture (Recovery 1). During the final 30 min of recovery, one of three experimental conditions was employed: (1) no pressure (Control); (2) lower body positive pressure (+45 mmHg; LBPP); or (3) lower body negative pressure (−20 mmHg; LBNP). Participants donned a loose‐fitting neoprene suit below the waist to minimize the effects of air flow and air was also circulated into the unsealed box during the Control condition to simulate air flow during the pressure conditions. The pressure was verified every minute manually by a pressure gauge. At the end of Recovery 1, participants were moved back to the upright seated cycle ergometer (<5 min) for a second 15 min exercise bout at 85% of their maximal oxygen consumption (Exercise 2). Finally, participants were transitioned back to the pressure box sealed at the waist (<5 min) for a second 45 min recovery period in the upright seated position (Recovery 2). The experimental condition employed during Recovery 1 was repeated during the final 30 min of Recovery 2. At the end of the experimental session, participants remained in the upright seated position for a period of local perfusion of sodium nitroprusside (50 mmol, Hospira, Lake Forest, IL) at 6 *μ*L·min^−1^ for ~25–30 min to determine maximum cutaneous blood flow. Perfusion continued until a stable plateau of cutaneous blood flow was achieved for at least 2 min. At this time mean arterial pressure was measured by manual auscultation for the determination of maximal cutaneous vascular conductance (CVC). A final nude body mass measurement and urine sample were obtained at the end of the experimental session.

### Measurements

Oesophageal temperature was measured continuously using a pediatric thermocouple probe of ~2 mm in diameter (Mon‐a‐therm, Mallinckrodt Medical, St Louis, MO) inserted through the nose ~40 cm past the entrance of the nostril. Mean skin temperature was calculated according to the proportions determined by Hardy and Du Bois (1938) based on local skin temperature at ten sites (forehead [7%], upper back [8.75%], chest [8.75%], bicep [9.5%], forearm [9.5%], abdomen [8.75%], lower back [8.75%], quadriceps [9.5%], hamstring [9.5%], and front calf [20%]). Temperature data were collected using a data acquisition module (model 3497A, Agilent Technologies Canada Inc., Mississauga, ON, Canada) at a sampling rate of 15 s and simultaneously displayed and recorded in spreadsheet format on a personal computer with LabVIEW software (version 7.0; National Instruments, Austin, TX).

Heart rate was recorded continuously, and stored every 15 s using a Polar coded WearLink and transmitter, Polar RS400 interface, and Polar Trainer 5 software (Polar Electro, Oy, Finland). Mean arterial pressure was estimated continuously before exercise and during each recovery using a Finometer (Finapres Medical Systems, Amsterdam, the Netherlands) and was measured by manual auscultation during the final minute of each exercise. Finometer measurements were derived from the beat‐to‐beat recording of the left middle finger arterial pressure waveform via the volume‐clamp method (Penaz 1973). The Finometer was calibrated using upper arm return‐to‐flow systolic pressure detection (Bos et al. 1996) and physiocal criteria (Wesseling et al. 1995) following brachial artery pressure reconstruction (Gizdulich et al. 1996, 1997). The right arm was supported at heart level for calibration as well as the measurement periods prior to and following each exercise.

The ventilated capsule technique was employed for the purpose of measuring local sweat rate. Forearm sweat rate was measured from 3.8 cm^2^ capsules attached to the skin with adhesive rings and topical skin glue (Collodion HV, Mavidon Medical Products, Lake Worth, FL). The sweat capsules were custom built to house the laser‐Doppler flow probe (see below) and allowed for the simultaneous measurement of local sweat rate and cutaneous blood flow. Compressed anhydrous nitrogen was passed through each capsule at a rate of 0.75 L·min^−1^. Long vinyl tubes were used to supply the dry gas to and from the ventilated capsules to ensure optimal equilibration with ambient environmental conditions for the experimental sessions. Water content of the effluent air was measured using capacitance hygrometers (Model HMT333, Vaisala, Woburn, WA). Forearm sweat rate was calculated using the difference in water content between the effluent and influent air multiplied by the flow rate and normalized for the skin surface area under the capsule (in mg·min^−1^·cm^−2^).

Forearm cutaneous blood flow was estimated at 32 Hz using laser‐Doppler velocimetry (PeriFlux System 5000, Perimed AB, Stockholm, Sweden). A laser‐Doppler probe integrated with a 7 laser array (integrating probe 413, Perimed AB) was placed directly over each microdialysis membrane and was secured for the entire experimental session. Cutaneous vascular conductance was subsequently calculated as the ratio of cutaneous blood flow (perfusion units) to mean arterial pressure and expressed as a percentage of maximum as determined during the maximal cutaneous blood flow protocol.

### Data analysis

In order to detect the mechanisms governing the baroreceptor mediated suppression in postexercise heat loss, CVC and sweat rate at each treatment site were calculated and presented at baseline, end of each exercise, and at 5 min intervals throughout recovery. Subsequently, CVC and sweat rate at the Ringer's site were compared to the l‐NAME, Theophylline, and Bretylium sites to evaluate the time‐dependent modulators of postexercise CVC and sweating. Additionally, CVC and sweat rate at each site were compared independently as a function of the three pressure conditions in order to evaluate the mechanisms underlying the baroreceptor‐mediated modulation of CVC and sweat rate. Secondary variables including oesophageal and mean skin temperatures, heart rate, and mean arterial pressure were presented as a 5 min average at baseline, end of each exercise (note only one measurement for mean arterial pressure), and every 5 min during each recovery period. It is important to note that for all variables in both recovery periods the time point at 5 min was omitted to account for the transition from the cycle ergometer to the pressure box (<5 min) and subsequent calibration of the Finometer (~3 min). Finally, maximal CVC was calculated as an average of cutaneous blood flow (perfusion units) once a plateau was achieved (~2 min), divided by the corresponding mean arterial pressure, and multiplied by 100 to be expressed as perfusion units per mmHg.

### Statistical analysis

A three‐way repeated measures analysis of variance (ANOVA) was used for CVC and sweat rate which were analysed with factors of time (nineteen levels: baseline, end of the first and second exercise, and min 10, 15, 20, 25, 30, 35, 40 and 45 of the first and second recovery), treatment site (four levels: Ringer's, l‐NAME, Theophylline, and Bretylium), and pressure condition (three levels: Control, LBPP, and LBNP). Cold pressor test CVC as well as that during the maximal vasodilation protocol were analysed with a separate two‐way ANOVA with a factor of treatment site (cold pressor test: two levels [Ringer's and Bretylium]; maximal CVC: four levels) and pressure condition (three levels). In addition, all secondary variables (oesophageal and mean skin temperatures, heart rate, and mean arterial pressure) were each analysed separately using a two‐way ANOVA with a factor of time (nineteen levels) and pressure condition (three levels). In order to ensure participants were similarly hydrated in each of the three experimental sessions, a two‐way ANOVA was conducted for urine specific gravity with factors of time (two levels: pre and post) and pressure condition (three levels) as well as a one‐way ANOVA with the factor of pressure condition (three levels) for pre‐mass and the percent change in body mass. Finally, a two‐way ANOVA with factors of exercise period (two levels) and pressure condition (three levels) was performed for external workload and the percentage of maximal oxygen consumption to ensure similar intensities were achieved during the final 10 min of each exercise. When a significant main effect was observed, post hoc comparisons were carried out using Student's paired samples *t*‐tests corrected for multiple comparisons using the Holm‐Bonferroni procedure. The level of significance for all analyses was set at an alpha level of *P *<**0.05. All statistical analyses were completed using the software package SPSS 21.0 for Windows (IBM, Armonk, NY). Values are presented as mean ± 95% confidence intervals unless otherwise indicated. Confidence intervals were calculated as 1.96 × standard error of the mean.

## Results

### Experimental sessions

Participants achieved a similar level of hydration for each experimental session as there was no main effect of pressure condition for pre nude body mass (*P *=**0.997) or for the percent change in body mass during the experimental sessions (*P *=**0.389). Urine specific gravity exhibited a main effect of time (*P *<**0.001) such that there was an increase from the start (Control: 1.011 ± 0.005; LBPP: 1.010 ± 0.005; LBNP: 1.012 ± 0.004) to the end (Control: 1.021 ± 0.005; LBPP: 1.020 ± 0.006; LBNP: 1.021 ± 0.003) of each experimental session. However, urine specific gravity did not differ as a function of pressure condition (*P *=**0.753). The percentage of maximal oxygen consumption attained was similar between Exercise 1 (Control: 85 ± 2%; LBPP: 85 ± 1%; LBNP: 85 ± 1%) and Exercise 2 (Control: 86 ± 2%; LBPP: 85 ± 1%; LBNP: 86 ± 1%, *P *=**0.544) and did not vary between pressure conditions (*P *=**0.822). Similarly, the external workload required did not differ between pressure conditions (*P *=**0.927) whereas it was lower during Exercise 2 (Control: 173 ± 34 W; LBPP: 175 ± 31 W; LBNP: 179 ± 31 W) compared to Exercise 1 (Control: 179 ± 33 W; LBPP: 184 ± 33 W; LBNP: 184 ± 31 W, *P *<**0.001).

### Cold pressor test

CVC at the Ringer's site was reduced following the cold pressor test to a similar extent in each pressure condition (Control: 22 ± 3%; LBPP: 23 ± 2%; LBNP: 22 ± 2%) compared to the corresponding baseline levels (Control: 33 ± 3%; LBPP: 34 ± 3%; LBNP: 32 ± 2%, *P *<**0.001). In contrast, baseline CVC at the Bretylium site (Control 32 ± 3%; LBPP: 34 ± 2%; LBNP: 34 ± 3%) did not differ from CVC at the end of the cold pressor test (Control: 32 ± 3%; LBPP: 34 ± 2%; LBNP: 33 ± 3%, *P *=**0.523).

### Hemodynamic responses

**Figure 1.**
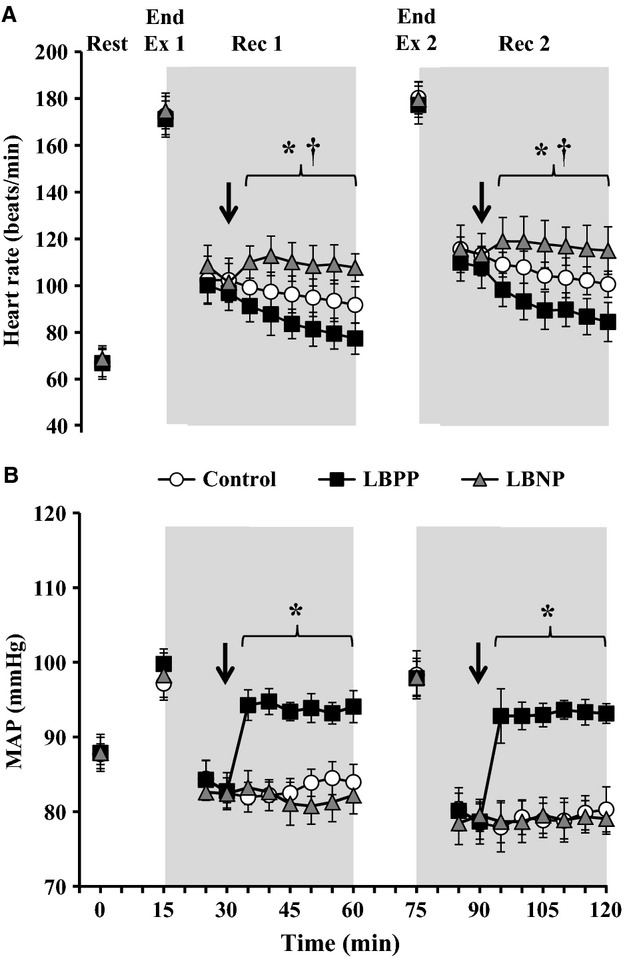
Heart rate (A) and mean arterial pressure (B; MAP) at baseline (Rest), end of exercise 1 and 2 (Ex 1 and Ex 2, respectively), and at 5 min intervals throughout the postexercise periods (Rec 1 and Rec 2) during a Control condition (i.e., no pressure; *open circles*), lower body positive pressure (LBPP;* black squares*) and lower body negative pressure (LBNP;* grey triangles*). Each pressure condition was employed at 15 min into recovery (indicated by a down arrow) and lasted for the final 30 min of each recovery. The values at 5 min of each recovery were omitted to allow for the transfer of participants to a pressure box sealed at the waist. Values are mean ± 95% confidence intervals. *LBPP significantly different from Control (*P *<**0.05); ^†^LBNP significantly different from Control (*P *<**0.05).

Heart rate responses are depicted in Fig. 1A. There was an interaction of time and pressure condition for heart rate (*P *<**0.001). Heart rate was similar between pressure conditions at baseline levels, at the end of each exercise, and up to 15 min into each recovery (all *P *>**0.100). However, the application of LBPP resulted in a progressive decrease compared to the Control condition by 14 ± 3 bpm and 16 ± 5 bpm at the end of Recovery 1 and 2, respectively. During the application of LBNP, heart rate was observed to increase compared to Control by 16 ± 5 bpm and 15 ± 4 bpm at the end of Recovery 1 and 2, respectively (*P *<**0.001).

Mean arterial pressure exhibited an interaction of time and pressure condition (*P *<**0.001; Fig. 1B). Baseline levels and end‐exercise measurements of mean arterial pressure were similar between pressure conditions (*P *>**0.354). Likewise, postexercise mean arterial pressure was reduced from baseline levels by 5 ± 0 and 9 ±1 mmHg in all conditions at 15 min into recovery following both Exercise 1 (*P *<**0.001) and Exercise 2 (*P *< 0.001) respectively. Noteworthy, the magnitude of postexercise hypotension was greater during Recovery 2 compared to Recovery 1 (all *P *<**0.020). In addition, mean arterial pressure was increased for the final 30 min of each recovery during LBPP compared to Control by 10 ± 1 mmHg (*P *<**0.001) whereas mean arterial pressure was not influenced by LBNP (all *P *>**0.119).

### Temperature responses

#### Oesophageal temperature

**Figure 2.**
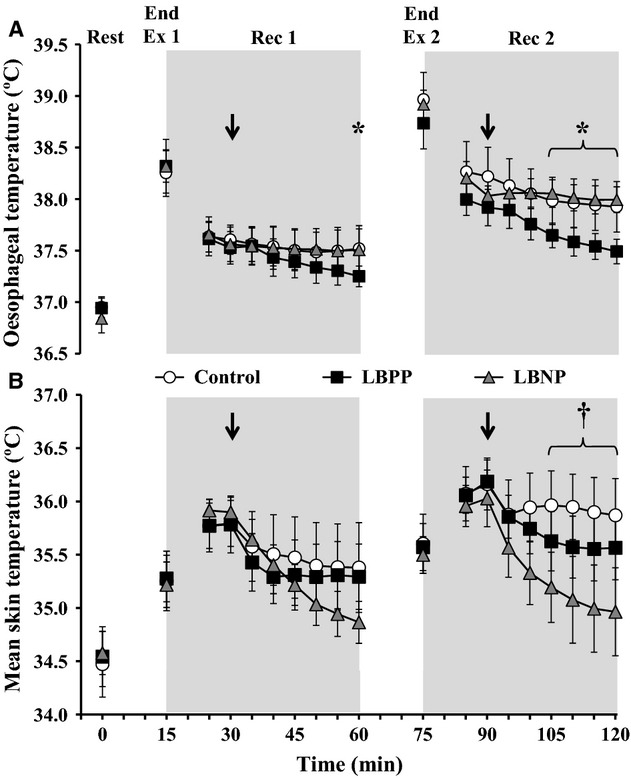
Oesophageal temperature (panel A) and mean skin temperature (panel B) at baseline (Rest), end of exercise 1 and 2 (Ex 1 and Ex 2, respectively), and at 5 min intervals throughout the postexercise periods (Rec 1 and Rec 2) during a Control condition (i.e., no pressure; *open circles*), and conditions applying lower body positive pressure (LBPP;* black squares*), and lower body negative pressure (LBNP;* grey triangles*). Each pressure condition was employed at 15 min into recovery (indicated by a down arrow) and lasted for the final 30 min of each recovery. The values at 5 min of each recovery were omitted to allow for the transfer of participants to a pressure box sealed at the waist. Values are mean ± 95% confidence intervals. *LBPP significantly different from Control (*P *<**0.05); ^†^LBNP significantly different from Control (*P *<**0.05).

An interaction of time and pressure condition was detected for oesophageal temperature (*P *<**0.001, Fig. 2A*)*. Specifically, no differences were found for oesophageal temperature between conditions at baseline levels (all *P *>**0.173), at the end of Exercise 1 (all *P *>**0.553) or at the end of Exercise 2 (all *P *>**0.077). In addition, oesophageal temperature was similar in the Control and LBNP pressure conditions throughout Recovery 1 (*P *=**0.898) and Recovery 2 (*P *=**0.754). Conversely, oesophageal temperature decreased more rapidly during the LBPP condition such that it was lower than Control by 0.27 ± 0.10°C at the end of Recovery 1 (*P *=**0.032) and for the final 20 min of Recovery 2 (all *P *<**0.020). Finally, oesophageal temperature was increased by 0.57 ± 0.15°C at the end of Exercise 2 compared to Exercise 1 in all pressure conditions (*P *<**0.001).

#### Mean skin temperature

Mean skin temperatures exhibited an interaction of time and pressure condition (*P *=**0.024, Fig 2B). Mean skin temperature was similar at baseline levels (all *P *>**0.117) as well as at the end of Exercise 1 and Recovery 1 (all *P *>**0.096). Although mean skin temperature remained similar between the Control and LBPP conditions until the end of Recovery 2 (all *P *>**0.075), it was progressively lower than Control during the final 20 min of Recovery 2 such that end‐recovery values were 0.83 ± 0.25°C lower in the LBNP condition (all *P *<**0.042).

### Heat loss responses

#### CVC – Effect of pressure

**Figure 3.**
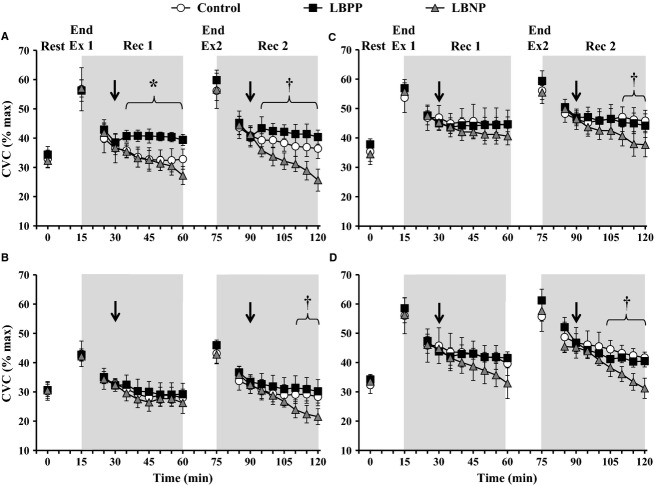
Cutaneous vascular conductance (CVC) presented as a percentage of maximum at baseline (Rest), end of exercise 1 and 2 (Ex 1 and Ex 2, respectively) and at 5 min intervals throughout the postexercise periods (Rec 1 and Rec 2) at skin sites perfused continuously with Lactated Ringer's solution (A), 10 mmol·L^−1^ *N*^*G*^‐nitro‐l‐arginine methyl ester (B; l‐NAME), 4 mmol·L^−1^ Theophylline (C), and 10 mmol·L^−1^ Bretylium Tosylate (D). Responses are presented during a Control condition (i.e., no pressure; *open circles*), and conditions employing lower body positive pressure (LBPP; *black squares*) and lower body negative pressure (LBNP;* grey triangles*). Each pressure condition was employed at 15 min into recovery (indicated by a down arrow) and lasted for the final 30 min of each recovery. The values at 5 min of each recovery were omitted to allow for the transfer of participants to a pressure box sealed at the waist. Values are mean ±95% confidence intervals. *LBPP significant different from Control (*P *<**0.05); ^†^LBNP significantly different from Control (*P *<**0.05).

An interaction of time and pressure condition was measured for CVC (*P *<**0.001, Fig. 3). CVC was similar between pressure conditions within all treatment sites at the end of Exercise 1 (all *P *>**0.251) and Exercise 2 (all *P *>**0.076). Similarly, CVC did not differ during Recovery 1 within the l‐NAME, Theophylline, and Bretylium sites (all *P *>**0.958), whereas CVC was increased from Control during the LBPP condition at the Ringer's site only for the final 30 min of Recovery 1 (all *P *<**0.028). Conversely, CVC at the Ringer's site was similar between LBPP and Control conditions throughout Recovery 2. The LBNP condition resulted in lower CVC for the last 15 min of Recovery 2 at each treatment site compared to the Control condition (all *P *<**0.011). Finally, there was no main effect of pressure condition (*P *=**0.904) for maximal CVC as determined at the end of each experimental session (Table 1).

**Table 1.** Absolute maximal cutaneous vascular conductance at each treatment as assessed at the end of each experimental session by infusion of 50 mmol·L^−1^ sodium nitroprusside via microdialysis at 6 *μ*L·min^−1^

	Control	LBPP	LBNP
Ringer's	217 ± 58	255 ± 102	235 ± 49
l‐NAME	199 ± 55	221 ± 40	218 ± 53
Theophylline	225 ± 67	244 ± 36	268 ± 82
Bretylium	256 ± 81	237 ± 55	245 ± 67

Lower body positive pressure condition, LBPP; Lower body negative pressure condition, LBNP; l‐NAME, *N*^*G*^‐nitro‐l‐arginine methyl ester. Values are mean ± 95% confidence intervals. Values were calculated from maximal cutaneous blood flow (perfusion units) averaged over 2 min, multiplied by 100 and divided by the corresponding mean arterial pressure. No main effect of treatment site (*P *=**0.157) or pressure condition (*P *=**0.904) was detected.

#### CVC – Effect of treatment site

An interaction of time and treatment was also measured for CVC (*P *<**0.001, Table 2). CVC was similar at baseline levels, as well as at the end of Exercise 1 and 2 at the Ringer's, Theophylline, and Bretylium sites in each pressure condition (all *P *>**0.136) whereas CVC at the l‐NAME site was lower than Ringer's at the end of each exercise period (all *P *<**0.001). During the Control condition, CVC at the Ringer's site paralleled that reported at the l‐NAME site throughout Recovery 1 (all *P *>**0.087) whereas CVC was elevated at the Ringer's site throughout Recovery 2 (all *P *<**0.002). In contrast, CVC at the Ringer's site was elevated during the LBPP and LBNP conditions relative to the l‐NAME site throughout Recovery 1 and 2 (all *P *<**0.023). Additionally, whereas CVC was reduced at the Ringer's site compared to the Theophylline and Bretylium sites throughout Recovery 1 and 2 of the Control and LBNP conditions (all *P *<**0.047), CVC was similar between these sites during the application of LBPP (*P *>**0.067). Finally, there was no main effect of treatment site detected for maximal CVC (*P *=**0.259, Table 1).

**Table 2.** Cutaneous vascular conductance responses for each pressure condition at each treatment site (Ringer's, *N*^*G*^‐nitro‐l‐arginine methyl ester [l‐NAME], Theophylline [Theo], and Bretylium) at baseline (Rest), end of each exercise (End‐Ex 1 and End‐Ex 2), and at 15 min intervals throughout each recovery (Recovery 1 and Recovery 2)

	Rest	End‐Ex 1	Recovery 1			End‐Ex 2	Recovery 2		
	0	15	30	45	60	75	90	105	120
Control (% Max)									
Ringer's	33 ± 3	57 ± 7	36 ± 5	33 ± 4	33 ± 3	56 ± 6	41 ± 3	38 ± 3	36 ± 3
l‐NAME	30 ± 3	43 ± 4*	33 ± 2	28 ± 3	28 ± 3	43 ± 4*	32 ± 3*	29 ± 4*	28 ± 3*
Theo	35 ± 3	54 ± 5	47 ± 4	46 ± 6	44 ± 5	56 ± 4	46 ± 3	46 ± 5	46 ± 4
Bretylium	32 ± 3	56 ± 6	46 ± 6	43 ± 4	40 ± 4	56 ± 5	47 ± 5	45 ± 3	42 ± 2
Lower body positive pressure (% Max)									
Ringer's	34 ± 3	56 ± 4	39 ± 3	41 ± 2	39 ± 2	60 ± 3	40 ± 3	42 ± 2	40 ± 2
l‐NAME	31 ± 3	43 ± 2*	32 ± 2*	30 ± 4*	29 ± 4*	46 ± 2*	33 ± 2*	31 ± 4*	30 ± 4*
Theo	38 ± 2	57 ± 3	45 ± 2*	44 ± 3	45 ± 3	59 ± 3	47 ± 2	46 ± 3	44 ± 4
Bretylium	34 ± 2	59 ± 4	44 ± 2*	43 ± 2	42 ± 2	61 ± 4	47 ± 3	41 ± 2	40 ± 2
Lower body negative pressure (% Max)									
Ringer's	32 ± 2	57 ± 3	37 ± 2	33 ± 3	27 ± 3	57 ± 4	41 ± 2	32 ± 3	26 ± 4
l‐NAME	31 ± 2	42 ± 2*	32 ± 2*	26 ± 3*	26 ± 4*	43 ± 3*	32 ± 3*	27 ± 2*	22 ± 3*
Theo	34 ± 4	56 ± 2	45 ± 3*	42 ± 2*	41 ± 3*	55 ± 2	46 ± 2*	42 ± 3*	38 ± 4*
Bretylium	34 ± 2	56 ± 3	45 ± 2*	39 ± 5*	33 ± 5*	58 ± 3	45 ± 2*	38 ± 3*	31 ± 3*

Values presented as mean ± 95% confidence interval.

* Different from Ringer's in the same pressure condition (*P *<**0·05).

#### Sweating – Effect of pressure

**Figure 4.**
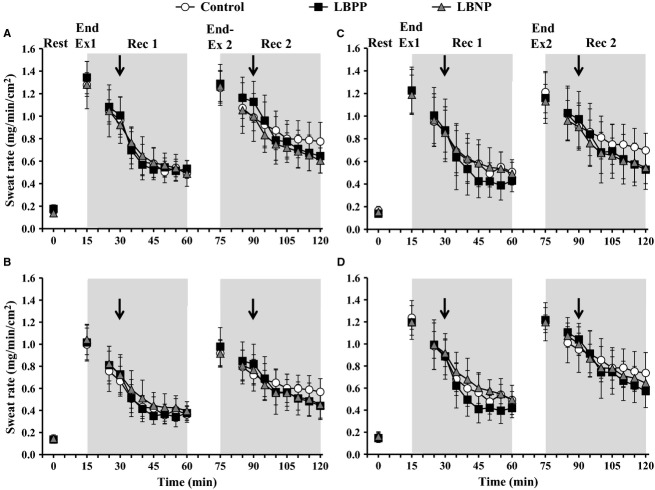
Local sweat rate presented at baseline (Rest), end of exercise 1 and 2 (Ex 1 and Ex 2, respectively) and at 5 min intervals throughout the postexercise periods (Rec 1 and Rec 2) at skin sites perfused continuously with Lactated Ringer's solution (A), 10 mmol·L^−1^ *N*^*G*^‐nitro‐l‐arginine methyl ester (B; l‐NAME), 4 mmol·L^−1^ Theophylline (C), and 10 mmol·L^−1^ Bretylium Tosylate (D). Responses are presented during a Control condition (i.e., no pressure; *open circles*), and conditions employing lower body positive pressure (LBPP; *black squares*) and lower body negative pressure (LBNP; *grey triangles*). Each pressure condition was employed at 15 min into recovery (indicated by a down arrow) and lasted for the final 30 min of each recovery. The values at 5 min of each recovery were omitted to allow for the transfer of participants to a pressure box sealed at the waist and. Values are mean ±95% confidence intervals. No differences between pressure conditions were found at any skin site throughout the experimental protocol (*P *>**0.05).

There was no interaction of time and pressure condition (*P *=**0.470) for sweat rate (Fig. 4). Specifically, sweat rate within each treatment site was not different between pressure conditions at baseline levels and remained similar between pressure conditions for the entire experimental session (*P *=**0.963). However, there was a main effect of time for sweat rate (*P *<**0.001) such that sweating increased during each exercise bout and decreased towards baseline levels during each recovery period.

#### Sweating – Effect of treatment site

Sweat rate did exhibit an interaction of time and treatment site (*P *<**0.001; Table 3). Specifically, sweat rate at the l‐NAME site was reduced from the Ringer's site in all pressure conditions at the end of Exercise 1 (all *P *<**0.018) and Exercise 2 (all *P *<**0.012). In addition, while sweat rate at the Ringer's site was similar to that at the l‐NAME site for the final 25 min of Recovery 1 (all *P *>**0.068), sweat rate throughout Recovery 2 was elevated at the Ringer's site (all *P *<**0.015) in all pressure conditions. Conversely, sweat rate did not differ throughout the experimental protocol in any pressure condition at the Theophylline or Bretylium sites compared to the Ringer's site (all *P *>**0.078).

**Table 3.** Local sweat rate responses for each pressure condition at each treatment site (Ringer's, *N*^*G*^‐nitro‐l‐arginine methyl ester [l‐NAME], Theophylline [Theo], and Bretylium) at baseline (Rest), end of each exercise (End‐Ex 1 and End‐Ex 2), and at 15 min intervals throughout each recovery (Recovery 1 and Recovery 2)

	Rest	End‐Ex 1	Recovery 1			End‐Ex 2	Recovery 2		
	0	15	30	45	60	75	90	105	120
Control (mg·min^−1^·cm^−2^)									
Ringer's	0.16 ± 0.05	1.28 ± 0.21	0.96 ± 0.20	0.58 ± 0.14	0.49 ± 0.11	1.25 ± 0.16	0.99 ± 0.21	0.80 ± 0.16	0.77 ± 0.17
l‐NAME	0.14 ± 0.03	1.00 ± 0.15*	0.66 ± 0.14	0.42 ± 0.10	0.36 ± 0.07	0.92 ± 0.13*	0.72 ± 0.14*	0.60 ± 0.13*	0.57 ± 0.12
Theo	0.17 ± 0.04	1.22 ± 0.19	0.87 ± 0.19	0.58 ± 0.17	0.51 ± 0.11	1.21 ± 0.18	0.91 ± 0.21	0.75 ± 0.18	0.70 ± 0.15
Bretylium	0.16 ± 0.05	1.24 ± 0.16	0.88 ± 0.16	0.56 ± 0.17	0.49 ± 0.13	1.20 ± 0.17	0.95 ± 0.21	0.78 ± 0.18	0.74 ± 0.19
Lower body positive pressure (mg·min^−1^·cm^−2^)									
Ringer's	0.18 ± 0.04	1.34 ± 0.14	1.01 ± 0.17	0.53 ± 0.07	0.53 ± 0.07	1.29 ± 0.17	1.13 ± 0.18	0.77 ± 0.15	0.65 ± 0.15
l‐NAME	0.14 ± 0.03	1.02 ± 0.16*	0.73 ± 0.18	0.35 ± 0.07	0.38 ± 0.07	0.98 ± 0.17*	0.82 ± 0.18	0.56 ± 0.16	0.44 ± 0.12
Theo	0.14 ± 0.03	1.23 ± 0.21	0.87 ± 0.28	0.42 ± 0.15	0.43 ± 0.10	1.16 ± 0.22	0.97 ± 0.25	0.69 ± 0.24	0.53 ± 0.17
Bretylium	0.15 ± 0.03	1.19 ± 0.15	0.89 ± 0.21	0.41 ± 0.13	0.42 ± 0.09	1.22 ± 0.11	1.04 ± 0.15	0.74 ± 0.20	0.57 ± 0.15
Lower body negative pressure (mg·min^−1^·cm^−2^)									
Ringer's	0.13 ± 0.03	1.27 ± 0.10	0.87 ± 0.14	0.55 ± 0.14	0.52 ± 0.08	1.25 ± 0.13	1.00 ± 0.12	0.75 ± 0.13	0.65 ± 0.13
l‐NAME	0.14 ± 0.04	1.06 ± 0.13*	0.70 ± 0.15	0.43 ± 0.11	0.44 ± 0.11	0.92 ± 0.11*	0.78 ± 0.13	0.58 ± 0.12	0.49 ± 0.14
Theo	0.16 ± 0.04	1.21 ± 0.17	0.81 ± 0.23	0.54 ± 0.21	0.55 ± 0.14	1.15 ± 0.15	0.92 ± 0.18	0.61 ± 0.16	0.52 ± 0.14
Bretylium	0.16 ± 0.03	1.22 ± 0.07	0.92 ± 0.11	0.60 ± 0.13	0.50 ± 0.07	1.20 ± 0.06	1.01 ± 0.09	0.79 ± 0.10	0.68 ± 0.11

Values presented as mean ± 95% confidence interval.

* Different from Ringer's in the same pressure condition (*P *<**0·05).

## Discussion

In this study, we evaluated the mechanisms involved in the baroreceptor‐mediated suppression of cutaneous blood flow and sweating during recovery from intermittent exercise. Consistent with our first hypothesis, LBPP was associated with increases in CVC from a Control condition and this increase was blunted by the non‐selective inhibition of nitric oxide synthase. Furthermore, CVC was reduced with LBNP which was not affected by inhibition of noradrenergic vasoconstriction. Conversely, we did not observe any baroreceptor‐mediated modulation of sweating during Recovery 1 or 2. However, a role for nitric oxide was detected during the early stages of Recovery 1 and throughout Recovery 2 as sweat rate at the Ringer's site was elevated from the l‐NAME site. Consistent with our second hypothesis, CVC did not differ between pressure conditions at the Theophylline or Bretylium sites thereby implicating a potential role for adenosine receptors and noradrenergic vasoconstriction in the baroreceptor‐mediated suppression of cutaneous blood flow. Finally, in line with our third hypothesis, we observed a temperature‐dependent influence of baroreceptor loading status on CVC shown by a blunted influence of LBPP during Recovery 2 relative to Recovery 1. Taken together, our findings indicate that baroreceptor loading status modulates cutaneous blood flow during postexercise recovery through nitric oxide, adenosine receptor, and noradrenergic vasoconstrictor‐dependent mechanisms; however, an influence of baroreceptors on postexercise sweating was not observed.

### Effects of pressure on postexercise hyperthermia

The influence of baroreceptor loading status on postexercise heat loss has been evidenced in numerous studies (Kenny et al. 2003a,b; Journeay et al. 2004; Gagnon et al. 2008). The reversal of baroreceptor unloading (i.e., blunting postexercise hypotension) has been shown to increase cutaneous blood flow and sweating (Journeay et al. 2004; Gagnon et al. 2008), and this has been shown to translate into a more rapid decrease in core body temperature (Journeay et al. 2004). Consistent with the study by Journeay et al. (2004), we observed a more rapid reduction in postexercise core temperature such that oesophageal temperature was ~0.5°C lower in the LBPP condition relative to the Control condition at the end of Recovery 2 (Fig. 2). On the other hand, it is important to note that the postexercise core temperature response was similar during the LBNP and Control conditions during Recovery 1 and 2. This is consistent with Journeay et al. (2004) and with our heat loss data (discussed below) as differences during LBNP were not found for sweating during Recovery 1 or 2 and CVC was only reduced in the later stages of Recovery 2.

### Effects of pressure on postexercise CVC

The effects of baroreceptor loading status on cutaneous blood flow have been well documented (Kellogg et al. 1990; Mack et al. 1995; Crandall et al. 1996; Journeay et al. 2004). In particular, it is believed that baroreceptor unloading associated with postexercise hypotension is involved in suppressing postexercise cutaneous blood flow (Kenny and Journeay 2010). Our findings are consistent with this notion such that LBPP application resulted in an increase in CVC at the Ringer's site during Recovery 1. In addition, we demonstrated that this response is largely nitric oxide‐dependent as no effect of LBPP was observed at the l‐NAME site (Fig. 3). However, it is important to note that this increase in CVC associated with LBPP was only observed in Recovery 1 as CVC was similar between the LBPP and Control conditions during Recovery 2. Oesophageal temperature was also ~0.5°C higher in the Control condition at the end of Recovery 2 compared to Recovery 1. Thus, the elevation in CVC at the Ringer's site during Recovery 2 is likely a thermal‐mediated response and is consistent with previous findings implicating a temperature‐dependent influence of baroreceptor loading status on CVC during the postexercise period (Gagnon et al. 2008). Interestingly, postexercise CVC was similar during Recovery 1 and 2 at the l‐NAME site, lending evidence to suggest that the mechanism involved in elevating CVC during Recovery 2 at the Ringer's site may also be nitric oxide‐dependent.

A previous study has implicated adenosine receptors and noradrenergic vasoconstriction as modulators of postexercise CVC (McGinn et al. 2014). Consistent with these findings, CVC was elevated in the present study throughout Recovery 1 and 2 at the Theophylline and Bretylium sites compared to the Ringer's site. Our observations extend the role for adenosine receptor and noradrenergic vasoconstrictor inhibition in modulating CVC during recovery following exercise in the heat as well as following repeated exercise bouts. Interestingly, we observed no effect of LBPP on CVC in Recovery 1 or 2 for the Theophylline or Bretylium sites. In fact, CVC at the Ringer's site was similar to CVC at the Theophylline and Bretylium sites during LBPP application. While the mechanism for these findings is unclear, some evidence has linked stress (e.g., pronounced hypotension or ischemia) to a greater release of ATP in the nucleus tractus solitarii in the rat (Van Wylen et al. 1988; St Lambert et al. 1997; Ichinose et al. 2012). This response has not been studied in humans; however, it is known that ATP is rapidly catabolized by ectonucleotidases to serve as the endogenous source of adenosine. Given that ATP is located in cutaneous sympathetic nerves (Burnstock 1990), and the substantial role shown for adenosine in modulating postexercise CVC (McGinn et al. 2014), it is plausible to suggest that marked baroreceptor unloading during the postexercise period results in a concomitant increase in cutaneous adenosine receptor activation in an effort to restore and/or sustain mean arterial pressure. While speculative, our observations at the Bretylium site may reflect a similar mechanism given that ATP is known to be co‐localized and released with noradrenenaline and neuropeptide Y (Burnstock 1990), albeit future research is needed to expand on these interactive mechanisms.

In the present study, a further reduction in mean arterial pressure during LBNP application in Recovery 1 and 2 was not observed which parallels findings by Journeay et al. (2004). However, we observed a higher heart rate throughout LBNP application during Recovery 1 and 2 compared to Control, thereby indicating a greater level of baroreceptor unloading (Fig. 1). Unlike LBPP application, we did not observe any differences in CVC at any treatment site during Recovery 1 of the LBNP condition which is also consistent with previous findings (Journeay et al. 2004). Conversely, we were able to detect a decrease in CVC associated with LBNP application at all treatment sites during Recovery 2 when the magnitude of hyperthermia and postexercise hypotension was greater. Although this is inconsistent with the baroreceptor influence on cutaneous blood flow being reduced at greater levels of hyperthermia (Gagnon et al. 2008), it seems that combining the stress of Exercise 2 (as indicated by a greater magnitude of hyperthermia and hypotension) with LBNP during Recovery 2 placed a greater demand for the redistribution of blood from the skin to the central cavity. In line with studies using passive heat stress (Kellogg et al. 1990; Crandall et al. 1996) it is likely that the baroreceptors induced a greater withdrawal of cutaneous active vasodilator activity as the magnitude by which CVC was reduced with LBNP was similar at the Ringer's and Bretylium sites. However, we also noted that this decrease in CVC during Recovery 2 was markedly suppressed at the Theophylline site (by ~42%). These findings are consistent with our above statement concerning the potential link between baroreceptor unloading and adenosine release; however, our data alone cannot confirm or deny this hypothesis.

### Effects of pressure on postexercise sweating

It is well established that sweating is the primary avenue for heat dissipation during exercise in the heat (Kenny and Journeay 2010; Shibasaki and Crandall 2010); however, sweat rate is suppressed in the early stages of recovery towards baseline levels (Kenny and Journeay 2010). While some studies indicate that this suppression may involve a baroreceptor‐mediated component (Kenny et al. 2003b; Journeay et al. 2004; Gagnon et al. 2008), other studies suggest that baroreceptor loading status does not impact sweating (Mack et al. 2001; Wilson et al. 2001, 2005). Our findings indicate that changes in baroreceptor loading status do not alter sweating during recovery from exercise in the heat as evidenced by a similar level of sweating during Recovery 1 and 2 in each pressure condition at the Ringer's site. These results are not consistent with those by Journeay et al. (2004) who previously employed LBPP and LBNP conditions during the postexercise period. However, it is important to consider that the level of sweating during recovery in the present study was ~0.3 mg·min^−1^·cm^−2^ greater, likely due to a combination of warmer ambient conditions and ~0.3–0.6°C higher oesophageal temperature measured in our study. On the other hand, we did report a lower oesophageal temperature in the LBPP condition and a lower mean skin temperature in the LBNP condition compared to the Control condition (reduced by ~0.5 and ~0.8°C, respectively). Thus, a similar level of sweating occurred between all pressure conditions despite differences in thermal input which may implicate to some extent, although not conclusively, a baroreceptor‐mediated component. On the other hand, we also show that inhibiting adenosine receptors or preventing the release of noradrenaline and neuropeptide Y from sympathetic nerves does not modulate sweating during or following exercise irrespective of baroreceptor loading status.

Recent studies have identified nitric oxide as a contributor to sweat production during exercise (Welch et al. 2009; Stapleton et al. 2014); however, its role was not evident during recovery as the level of sweating was similar between the l‐NAME and Ringer's sites (Stapleton et al. 2014). Our findings are consistent with these prior reports during Exercise 1 and 2 such that we observed a reduction in sweating at the l‐NAME site relative to the Control site by ~0.2 mg·min^−1^·cm^−2^. However, unlike the results from Stapleton et al. (2014), we did demonstrate a role for nitric oxide during recovery. Specifically, we found sweat rate at the Ringer's site to be elevated from the l‐NAME site during the first 20 min of Recovery 1 and for the duration of Recovery 2. Sweat rate did not vary between pressure conditions which suggests a baroreceptor‐mediated modulation of sweating is unlikely. In fact, Gagnon et al. (2008) indicated that baroreceptor modulation of sweating during the postexercise period was only apparent in the later stages of recovery when core temperature was ≤0.6°C above baseline levels. Thus, at a level of hyperthermia exceeding 0.6°C above baseline levels, thermal input appeared to override any baroreceptor‐mediated input. Given that postexercise oesophageal temperature in the present study did not drop below this threshold (~0.6 and ~1.0°C above baseline at the end of Recovery 1 and 2 respectively), it seems that the elevated sweat rate at the Ringer's site was the result of thermal input that was nitric oxide‐dependent. Taken together, our findings suggest that nitric oxide can modulate sweating during the postexercise period, but only at higher core temperatures.

### Perspectives

In the present study we demonstrated a role for nitric oxide in modulating postexercise cutaneous blood flow and sweating. It is well known that there are three isoforms of nitric oxide synthase (NOS) found in humans (i.e., neuronal [nNOS], endothelial [eNOS], and inducible [iNOS]); however, due to the non‐selective inhibitory nature of l‐NAME, we were unable to decipher which enzyme isoform is responsible for our observations. A recent study by Kellogg et al. (2008) reported that nNOS was responsible for nitric oxide generation during centrally mediated cutaneous active vasodilation during whole‐body heat stress. In addition, it was postulated in this study that eNOS was not involved in the centrally‐mediated active vasodilatory response (Kellogg et al. 2008), whereas any role of iNOS was ruled out as it is typically found in such small concentrations in human skin (Wang et al. 1996). Given that baroreceptor loading status is believed to represent a central modulation of heat loss (Kenny and Journeay 2010), nNOS is the most probable isoform to account for the nitric oxide‐dependent modulation of cutaneous blood flow and sweating during the postexercise period.

Finally, it is important to note that although we induced marked changes in baroreceptor loading status during Recovery 1 as evidenced by heart rate and mean arterial pressure responses (Fig. 1), these cardiovascular as well as core body and skin temperature responses during Exercise 2 were similar between pressure conditions. Taken together, our results indicate that irrespective of any intervention applied to augment postexercise heat loss (i.e., lower body positive pressure application), the cardiovascular and body temperature responses to a subsequent exercise are unchanged. Considering that the progressive increase in the level of hyperthermia during repeated exercise bouts is reported to result from a sustained suppression of heat loss during the postexercise periods (Kenny and Gagnon 2010), employing such interventions to elevate heat dissipation during recovery would be critical to prevent heat‐related illness and/or injury. Specifically with regards to athletic and workplace conditions, such convenient and practical interventions (e.g., passive recovery such that the limbs are passively taken through the full range of motion and/or compression garments) need to be established and implemented in order to minimize the risk for adverse heat events.

## Conclusion

In summary, the present study indicates that the postexercise suppression of cutaneous blood flow is modulated by baroreceptor loading status. Our findings implicate a nitric oxide‐mediated component to postexercise vasodilation associated with LBPP; however, the involvement of adenosine receptors and noradrenergic vasoconstriction in contributing to this response cannot be discounted. In addition, the exacerbated suppression of cutaneous blood flow associated with LBNP was likely mediated by withdrawal of active vasodilation as we demonstrated this response to be intact with the inhibition of noradrenergic vasoconstriction. Conversely, we did not observe a baroreceptor‐mediated suppression of sweating during the postexercise period. However, it is possible that the level of hyperthermia during recovery was such that thermal afferents superseded any influence of baroreceptors. Finally, we also found this thermal‐mediated response of sweating during recovery to exhibit a nitric oxide‐dependent component.

## Acknowledgments

The authors are indebted to the participants who volunteered for the present study as well as to Pegah Akbari for her assistance with data collection. The authors would like to thank Mr. Michael Sabino of Can‐Trol Environmental Systems Limited (Markham, ON, Canada) for his support.

## Competing Interests

None declared.
